# Associations of Gait, Cognitive Imparment, Dementia and Obstructive Sleep Apnea inIndividuals with Down Syndrome: Preliminary Findings

**DOI:** 10.1002/alz.094955

**Published:** 2025-01-09

**Authors:** Aline de Souza Gonçalves Gomes da Conceição Conceição, Ana Paula Assunção Cecilio, Livea Carla Fidalgo Garcêz Sant´Ana, Luciana Fonseca, Orestes Vicente Forlenza

**Affiliations:** ^1^ Department and Institute of Psychiatry, Hospital das Clínicas da Faculdade de Medicina da Universidade de São Paulo HCFMUSP, São Paulo, São Paulo Brazil; ^2^ Laboratory of Neuroscience (LIM‐27), Department and Institute of Psychiatry, Faculty of Medicine, University of São Paulo, São Paulo, São Paulo Brazil; ^3^ Laboratory of Neuroscience (LIM‐27), Department and Institute of Psychiatry, Faculty of Medicine, University of São Paulo, São Paulo‐SP, São Paulo Brazil; ^4^ Faculty of Medicine, University of São Paulo‐ Brazil., São Paulo Brazil; ^5^ Washington State University, Spokane, WA USA; ^6^ Faculdade de Medicina da Universidade de São Paulo, São Paulo, São Paulo Brazil

## Abstract

**Background:**

It is known that atipical aging in DS is related to a high risk of early dementia, with neuropathological charactheristics compatible with Alzheimer´s disease (AD). Changes in functional mobility are expected throughout the aging process, with impairments in motor performance, involving balance and gait. Growing evidence suggests that sleep disruption may also accelerate the progression to symptomatic Alzheimer’s disease (AD) in this population. Therefore, it is mandatory to assess sleep quality and diagnose sleep‐disordered breathing, especially obstructive sleep apnea (OSA), associated with DS, to improve cognition and quality of life, preventing related comorbidities.

**Method:**

We evaluated 66 individuals with DS (≥20 years), divided into 3 groups: stable cognition, prodromal dementia, and Alzheimer’s disease. Each individual was evaluated with the Performance‐Oriented Mobility Assessment (POMA) protocol, Polysomnography, and Cambridge Examination for Mental Disorders of Older People with Down’s Syndrome and Others with Intellectual Disabilities interview adapted for adults with DS (CAMDEX‐DS).

**Result:**

The logistic regression model with the stepwise backward technique, with the initial model including age, gender, body mass index (BMI), degree of intellectual disability, and apnea‐hypopnea index (AHI), showed that gait performance in POMA are independent factors associated with the diagnosis of control and dementia (POMA p < 0.005). All cognitive domains were correlated with OSA, but only orientation had a significant correlation (p‐value 0.268). We did not find a difference in OSA scores among POMA.

**Conclusion:**

The POMA results indicate that those with AD have worse gait performance and a higher risk of falls relative to those with dementia and stable cognition. There was no evidence of correlation between gait speed and OSA, which may have been influenced by the sample size (n:17). Further longitudinal studies with larger samples are needed to confirm our results.

